# Loss of Wiz Function Affects Methylation Pattern in Palate Development and Leads to Cleft Palate

**DOI:** 10.3389/fcell.2021.620692

**Published:** 2021-06-02

**Authors:** Ivana Bukova, Katarzyna Izabela Szczerkowska, Michaela Prochazkova, Inken M. Beck, Jan Prochazka, Radislav Sedlacek

**Affiliations:** ^1^Laboratory of Transgenic Models of Diseases and the Czech Centre of Phenogenomics, Institute of Molecular Genetics of the Czech Academy of Sciences, Prague, Czechia; ^2^Animal Research Centre, Ulm University, Ulm, Germany

**Keywords:** Wiz, G9a/GLP, histone methylation, cleft palate, development, craniofacial

## Abstract

WIZ (Widely Interspaced Zinc Finger) is associated with the G9a-GLP protein complex, a key H3K9 methyltransferase suggesting a role in transcriptional repression. However, its role in embryonic development is poorly described. In order to assess the loss of function of WIZ, we generated CRISPR/Cas9 WIZ knockout mouse model with 32 nucleotide deletion. Observing the lethality status, we identified the WIZ knockouts to be subviable during embryonic development and non-viable after birth. Morphology of developing embryo was analyzed at E14.5 and E18.5 and our findings were supported by microCT scans. Wiz KO showed improper development in multiple aspects, specifically in the craniofacial area. In particular, shorter snout, cleft palate, and cleft eyelids were present in mutant embryos. Palatal shelves were hypomorphic and though elevated to a horizontal position on top of the tongue, they failed to make contact and fuse. By comparison of proliferation pattern and histone methylation in developing palatal shelves we brought new evidence of importance WIZ dependent G9a-GLP methylation complex in craniofacial development, especially in palate shelf fusion.

## Introduction

Cleft palate is one of the most common congenital defects observed at birth. Palatogenesis and secondary palate formation is a complex process that entails multiple events including growth, elevation, and fusion of palatal shelves. The palatal shelves consist mostly of neural crest cells derived mesenchyme (Ito et al., 2003). Palate formation is initiated around E11 in mice when primordia of palatal shelves emerge from the inner part of the maxillary processes. From E12, palatal shelves start growing vertically alongside the tongue, then elevate to a horizontal position above the tongue around E14. Further growth ensures that palatal shelves on both sides are able to meet and fuse (Gritli-Linde, 2007; Bush and Jiang, 2012; Kim et al., 2017).

There are multiple publications providing insight into the cellular processes underlying palatal formation and its defects. One of the various proposed mechanisms regulating palatal outgrowth appears to be coordination of epithelial-mesenchyme interactions by Shh (Lan and Jiang, 2009) and Fgf (Rice et al., 2004) signaling pathway crosstalk, by Bmp (Zhang et al., 2002) and Tgf-β signaling (Nakajima et al., 2018). The proteolysis of extracellular matrix proteins (Enomoto et al., 2010) or epigenetic regulation such as DNA methylation (Kuriyama et al., 2008) have also been reported to contribute to palate formation.

One of the major methyltransferase mechanisms described to date represents G9a (Ehmt2- euchromatic histone lysine N-methyltransferase 2) and GLP (Ehmt1- euchromatic histone lysine N-methyltransferase 1) forming a heterodimeric complex. G9a and GLP deficiency leads to a dramatic reduction of mono- and dimethylated H3-K9 in ES cells, as well as severe growth retardation and embryonic lethality around day E9.5 (Tachibana et al., 2002, 2005). Wiz (Widely Interspaced Zinc Finger) was initially identified as a novel partner for this complex in various human and mouse cells types, including mouse ES cells. It was proposed WIZ helps with stability and specificity of this histone methylation complex (Ueda et al., 2006). Recently it was shown that WIZ and ZNF644, which both contain multiple zinc finger motifs, mediate the recruitment of the G9a/GLP histone methyltransferase complex to the specific gene loci by recognizing the DNA sequence and target H3-K9 methylation (Bian et al., 2015). The Wiz*^*MommeD*30^* mutant model was previously prepared by ENU (N-ethyl-N-nitrosourea) mutagenesis providing insight in its function. Daxinger et al. (2013) described developmental delay and embryonic lethality of Wiz*^*D*30/D30^* embryos between E10.5 and E12.5. WIZ has been proposed to share binding sites with transcriptional factor CTCF and to work as a transcriptional activator in neural tissue and necessary for normal behavior in mice (Isbel et al., 2016). Recently it was shown that WIZ forms a complex with CTCF and cohesin regulating DNA loop architecture and cell identity gene transcription (Justice et al., 2020).

**FIGURE 1:**
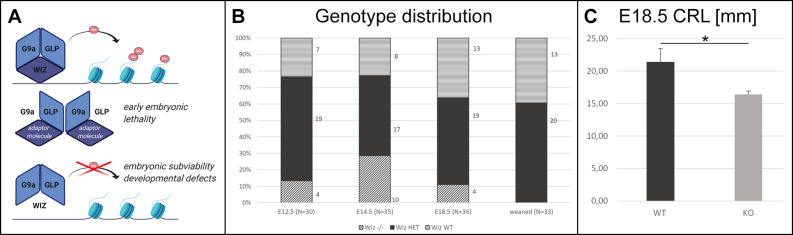
Wiz deficiency leads to developmental defects, growth retardation, and embryonic subviability. **(A)** Schematic cartoon visualizing hypothesized function of Wiz as an adaptor molecule and important member of G9a/GLP histone methylation complex. G9a and GLP are core members of this complex allowing the histone methylation. Both G9a and GLP are essential for embryonic development and viability (Tachibana et al., 2002, 2005), and we propose that Wiz deficiency causes improper complex assembly resulting in developmental defects and embryonic subviability caused by altered histone methylation. Created with BioRender.com. **(B)** Visual representation of genotype distribution in embryos of various developmental stages—E12.5 (N = 30), E14.5 (*N* = 35), E18.5 (*N* = 36), and weaned mice (*N* = 33). **(C)** Graph with embryonic Crown Rump Length (CRL) in mm. *Wiz^–/–^* exhibit substantial growth retardation compared to WT, *p* < 0, 05. Data are shown as mean ± SD. *p < 0.05.

Contrary to *G9a* and *GLP* KO (Tachibana et al., 2002, 2005), as shown in this study, functional ablation of Wiz in our case does not cause early embryonic lethality, although embryonic lethality becomes observable around E18.5, culminating in perinatal lethality of all *Wiz^–/–^* individuals. Those that survive until birth exhibit cases of cleft palate and other defects in the orofacial area. *Wiz* is highly expressed in the developing palate, especially in the epithelial layer of palatal shelves, corresponding to expression of the G9a and GLP. We investigated the effect of Wiz deficiency on the palatogenesis process. The morphological changes of palatal shelves were accompanied by a decrease in H3-K9 methylation marks in palatal shelf epithelium, however, the general proliferation pattern in palatal shelves was not affected. We conclude that the role of Wiz is pivotal to methylation complex G9a/GLP, specifically in medial epithelium in palatal shelves where it is likely to be involved in epigenetic regulation of proper timing of palatal shelves horizontalization during palatogenesis (Figure 1A).

## Materials and Methods

### Animals

Wiz knockout mouse model on a C57BL/6N background (Charles River Laboratories) used in this study was generated by targeting Wiz gene within a zinc-finger domain in exon 4 (transcript Wiz-001 ENSMUST00000087703) for frame-shift mutation by using CRISPR/Cas9 technique at Institute of Molecular Genetics, Prague. This exon is in all transcript variants except Wiz-009 ENSMUST00000170603.2, a short variant not containing any functional domain of the protein. Selected gRNAs for microinjections had following sequences: WIZ forward 5′-TGTAATACGACTCACTATA**GGCCTGCTTTGAGACACGAA** **A**GTTTTAGAGCTAGAAATAGC-3′ and WIZ reverse 5′-TGTAATACGACTCACTATA**GGCCGCAGATGTGAACGTGC** **G**GTTTTAGAGCTAGAAATAGC-3′. gRNAs were introduced to the fertilized oocytes of C57BL/6N strain and transferred into pseudo pregnant foster mice. Overall 15 transfers were done, 8 pups born and analyzed using PCR, gel electrophoresis, and sequencing. In total 2 F0 mice displayed deletions in targeted sequence: animal 79874 (del 32 nt + mismatch of 4 nt) and animal 79877 (del 3 nt/del 9 nt in two alleles). Animal 79874 with 32 nucleotide deletion in exon 4 was chosen as a founder. Genotyping was performed using following primers: forward: 5′-CTTCTCTGAGCCTCAGTTTCC-3′, reverse: 5′-GATGGCTTTGTTGACAGCAGG-3′ with Ta 64°C. Heterozygous mice were bred in order to obtain the homozygous embryos. The day at which a vaginal plug was present was considered as day 0.5 of pregnancy (gestational/embryonic day E0.5). Female mice were sacrificed and embryos harvested at days E13.5, E14.5, E15.5, and E18.5. Yolk sacks were collected for genotyping and embryos were fixed in 4% Paraformaldehyde (PFA) for at least 24 h depending on the following procedure.

### Embryo Viability

After no knockout offspring were successfully weaned from het × het crosses, embryo viability was assessed by systematic harvesting of embryos at E12.5, E14.5, and E18.5, observing them and genotyping. In total, 4 litters at E12.5 (30 embryos), 6 litters at E14.5 (35 embryos), and 6 litters at E18.5 (36 embryos) were harvested.

### MicroCT—Samples Preparation and Scanning

Whole mount embryos were fixed for 7 days in 4% PFA. Samples were then processed for microCT scanning including incubation in contrast agent. Samples were stained for at least 10 days (E14.5) and 2 weeks (E18.5) with Lugol’s Iodine solution. Stock solution (10 g KI and 5 g I2 in 100 ml H_2_O) was diluted to a 25% working solution in H_2_O to achieve neutral osmotic pressure and avoid tissue distortion. SkyScan 1272 high-resolution microCT (Bruker, Belgium) was set up for voxel size 2–3, 5 μm, and 1 mm Al filter. A 360° scan with 0.200° rotation step and three frames averaging setup was used for scanning. In total five *Wiz^–/–^* and five WT embryos were scanned at E18.5 and four *Wiz^–/–^* and two WT embryos at E14.5. Crown Rump Length (CRL) was measured on five Wiz*^–/–^* and five WT E18.5 embryos using ImageJ software. Frontal midline section from 3D reconstruction was used for the measurement.

### Histology

Whole embryos were fixed for 24 h in 4% PFA and further processed for paraffin embedding. The 10 μm thick frontal sections through the palate area were mounted on Superfrost Plus slides. The sections were then deparaffinized in 100% Xylene and rehydrated using an alcohol range (100%–70%–30%) for immunofluorescence or *in situ* hybridization staining.

For the immunofluorescence staining, frontal paraffin sections from E13.5–E15.5 Wiz WT and Wiz KO embryos were deparaffinized, antigen retrieval was performed with HIER Citrate buffer pH6 (Zytomed) for 15 min at 110°C. Sections were washed with Phosphate-buffered saline (PBS), permeabilized in 0.1% Triton-X at room temperature, and blocked with 2, 5% ready to use Normal Goat Serum (Vector Laboratories) for 1 h. Primary antibodies were diluted in EnVision FLEX Antibody Diluent (Agilent) and applied overnight at 4°C. Primary Ab Anti-Histone H3 (mono methyl K9) antibody—ChIP Grade (Abcam, ab9045) was used at 1:250 dilution and Anti-Histone H3 (di methyl K9) antibody (mAbcam 1220)—ChIP Grade (Abcam, ab1220) at 1:250 dilution. Primary Ab WIZ Antibody (Novus Biologicals, NBP1-80586) was used at 1:200 dilution. Following PBS wash, secondary antibodies were applied to sections for 1 h. Donkey anti-Rabbit IgG (H + L) Highly Cross-Adsorbed Secondary Antibody, Alexa Fluor 488 (Thermo Fisher Scientific, A-21206) was used at dilution 1:1,000 as well as Goat anti-Mouse IgG (H + L) Highly Cross-Adsorbed Secondary Antibody, Alexa Fluor Plus 594 (Thermo Fisher Scientific, A32742). Sections were mounted with Dako Fluorescence Mounting Medium (Agilent) and kept in 4°C until microscopic images were taken in Axio Image Z2.

### Quantification of Spectral Intensity on Immunofluorescence Sections

The spectral quantification of intensity of staining was performed in ImageJ software (version: 1.53h). The area was selected by “segmented line” tool and the spectral intensity for each channel (green, red) was quantified by “plot profile” tool. The values were processed in “R” for plot generation. Y-axis labels the arbitrary unit of signal intensity, X-axis labels the distance in medio-lateral direction in μm. *n* = 3 *Wiz^–/–^* and three WT samples, three sections each.

### *In situ* Hybridization

Digoxygenin-labeled RNA probes (DIG RNA labeling kit; Roche) were prepared by *in vitro* transcription from linearized pGEM^®^-T Easy (Promega, A1360) plasmids containing murine *G9a*, *GLP*, and *Wiz. G9a*, *GLP*, and *Wiz* sequences were obtained by PCR amplification of cDNA (murine muscle, kidney) and sub-cloned into the pGEM^®^-T Easy Vector. Sequence identity was verified by sequencing. Wiz containing plasmid was linearized by *Sac*II. G9a containing plasmid was linearized by *Sac*II and GLP containing plasmid by *Spe*I enzymes (Supplementary Figures 1A–C). We also generated Wiz, G9a, and GLP sense probes as a negative control (Supplementary Figure 1D). *In situ* hybridization was performed according to standard protocols (Wilkinson and Nieto, 1993) on frontal sections from E13.5, E14.5, and E15.5 embryos. Sections were mounted with Aquatex (Merck). We analyzed the expression pattern in forming palate and surrounding tissue.

### EdU Cell Proliferation Analysis

Pregnant females with embryos dedicated for proliferation analysis were injected peritoneally with 100 μl of 6.25 mM EdU (5-ethynyl-2′-deoxyuridine) solution in PBS each 90 min before the embryo harvest. Embryos were as usual fixed for 24 h in 4% PFA and processed for paraffin embedding and sectioning. The Click-iT EdU Cell Proliferation Kit for Imaging (Thermo Fisher Scientific) was used to visualize proliferating cells. Nuclear counterstain was performed with DAPI at 1:1,000 dilution. Cell counting for quantification was performed in ImageJ software on five sections from anterior and five sections from posterior region of palatal shelves from three *Wiz^–/–^* and three WT embryos at E13.5 and E14.5. For statistical comparison, linear mixed model was used with animal ID as a random effect.

Supplementary Methods related to SDS-PAGE and immunoblotting and Quantitative real time reverse transcription polymerase chain reaction (qRT-PCR) can be found in Supplementary Methods.

## Results

### Generation of Wiz Deficient Mouse Model

Targeted deletion of 32 nucleotides in exon 4 of Wiz causes frame-shift mutation and premature stop codon eliminating Wiz protein production (Supplementary Figure 2A). Western blot analysis using Anti-Wiz Ab detected a Wiz protein sized around 130 kDa in WT sample and lower amount of Wiz protein in heterozygous sample. No Wiz protein was detected in *Wiz^–/–^* embryonic sample (Supplementary Figure 2B). Anti-Wiz antibody shows high cross-reactivity. Mass spectrometry analysis of gel cutout (Supplementary Figure 2C) was performed to confirm the absence of Wiz protein in *Wiz^–/–^* protein lysate (Supplementary Figure 2D).

### Wiz Deficiency Causes Growth Retardation and Craniofacial Defects

No viable *Wiz^–/–^* mice were found after weaning. Moreover, no knockout pups were observed during the period before weaning. This finding strongly suggested that there is a late embryonic/perinatal lethal phenotype in *Wiz^–/–^* mice. We proceeded with a standardized search (Dickinson et al., 2016) for the embryonic lethal period, showing that genotype distribution was variable but relatively normal at all developmental stages until E18.5, even though we observed dead Wiz*^–/–^* embryos already from stage E12.5. After the birth, we were not able to recognize any viable *Wiz^–/–^* newborns, suggesting that *Wiz^–/–^* were subviable at E18.5 and show complete perinatal lethality (Figure 1B). This development corresponds to strong growth retardation at E18.5, which results in almost 25% lower CRL before birth compared to WT embryos (Figure 1C).

**FIGURE 2:**
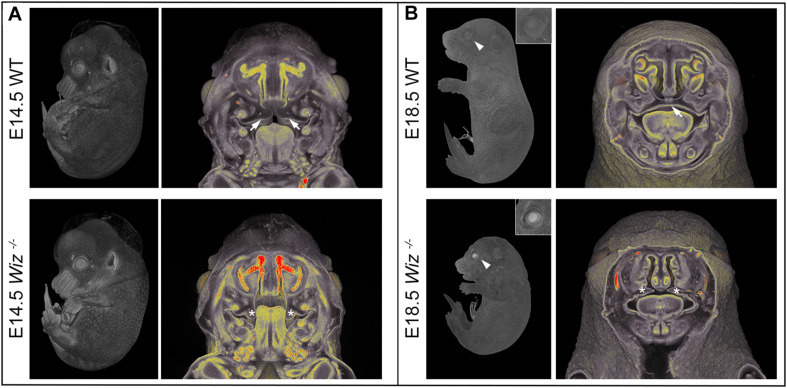
Wiz deficiency causes improper development especially in craniofacial area. **(A)** MicroCT images of E14.5 embryos. White arrows point to palatal shelves of WT embryo in proper horizontal position on top of the tongue. White asterisks indicate palatal shelves of *Wiz^–/–^* embryo remaining in vertical position along the tongue. **(B)** MicroCT images of E18.5 embryos. White arrowhead points to the eyelids, properly developed in WT and cleft in *Wiz^–/–^* embryo. Detail of the eye is showed in the top right corner. White arrow points to fully fused and formed palate of WT embryo. White asterisks indicate severely hypomorphic palatal shelves of Wiz KO embryo.

Besides the obvious growth retardation at E18.5 we observed improper development at multiple sites, especially in the craniofacial area as early as embryonic stage E14.5. At this stage, the horizontalization was delayed in *Wiz^–/–^* palatal shelves which remained lateral to the tongue (Figure 2A). Horizontalization of shelves was never observed even at E15.5 (five embryos out of five *Wiz^–/–^* embryos), when all WT palates were already fused. At E18.5 the craniofacial defect became even more visible with shorter snout morphology and also underdevelopment of eyelids. From the 12 *Wiz^–/–^* embryos harvested at E18.5 approximately 60% (7) showed full cleft palate and 40% (5) showed incomplete palatal shelves fusion. Palatal morphology at this stage implies that the shelves were later able to proceed to horizontalization; however, the delay of this process resulted in cleft phenotype (Figure 2B).

### WIZ Is Expressed in Palatal Shelves and Overlaps With G9a/GLP in Developing Palate Epithelium

**FIGURE 3:**
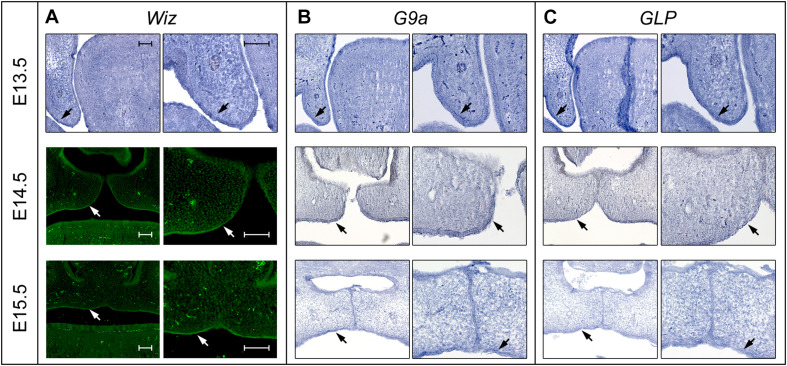
Expression pattern of Wiz in epithelia of palatal shelves overlaps with G9a/GLP. **(A)** *In situ* hybridization on palatal sections and immunofluorescent stained palatal sections of E13.5 WT (upper panels), E14.5 (middle panels), and E15.5 (lower panels) palatal sections showing strong expression of Wiz in the epithelia on oral side of palatal shelves (black and white arrows). **(B,C)** *In situ* hybridization on palatal sections of E13.5 WT (upper panels), E14.5 (middle panels), and E15.5 (lower panels) showing overlapping pattern of G9a/GLP expression with Wiz (black arrows). Scale bar represents 100 μm and applies for all images.

In order to investigate whether Wiz protein has a direct role in palatogenesis, we proceeded with fluorescent immunostaining of Wiz protein and *in situ* hybridization on histological sections of developing palate at E13.5 and E14.5. The nuclear localization of Wiz was indeed detected in palatal shelves, both in neural crest derived mesenchyme and in palatal shelf epithelium. Staining was stronger at the epithelial layer on the lateral side, which later forms the roof of oral cavity (Figure 3A, arrows). We also analyzed the expression of *G9a* and *GLP* to provide evidence that Wiz manifests its function during palate development via a possible interaction with the G9a and GLP methylation complex. Both *G9a* and *GLP* showed an overlapping expression pattern with *Wiz* and an increased expression at the oral side of palatal shelves (Figures 3B,C).

### Wiz Deficiency Does Not Affect Cell Proliferation but Suppresses Methylation Pattern in Developing Palate While Involved Signaling Pathways Are Unaffected

**FIGURE 4:**
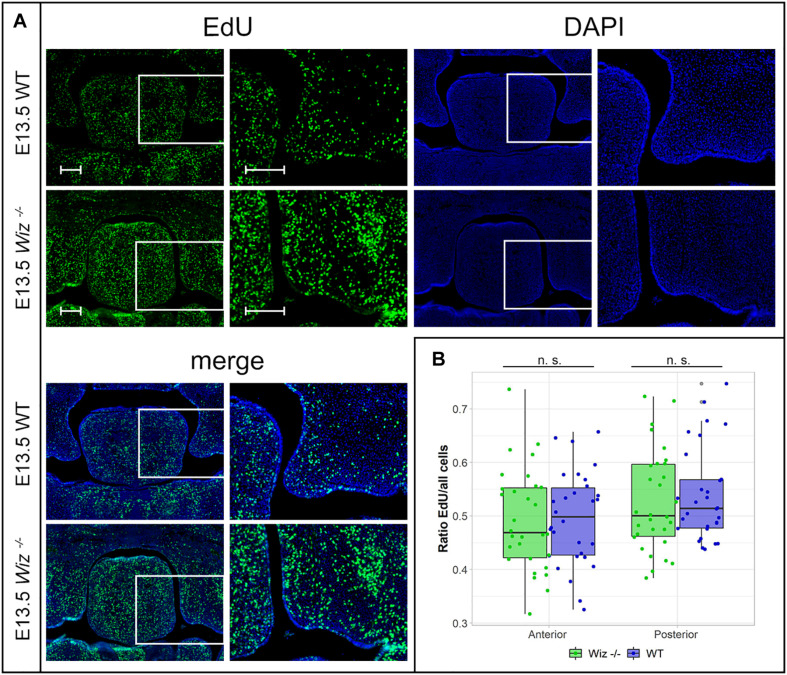
Wiz deficiency does not affect number of proliferative cells. **(A)** EdU labeling shows no difference in proliferation levels in palatal shelves between WT (upper panels) and KO (lower panels) palatal sections at E13.5. White rectangles represent the region magnified in the images on the left site, respectively. Scale bar represents 100 μm and applies for all images. **(B)** Quantification of EdU labeled proliferating cells shows no difference in ratio of proliferating between genotypes at E13.5 in anterior (*p* = 0.999) and posterior regions (*p* = 0.977) of palatal shelves. *n* = 3 *Wiz^–/–^* vs. 3 WT embryos, with five sections from anterior and five sections from posterior part of shelves for each embryo.

In connection to observed failure of horizontalization and fusion of palatal shelves at E15.5, we asked whether the phenotype might be caused by a reduction in proliferative capacity of cells in the developing palate at earlier stages (E13.5 and E14.5). After a short EdU pulse (90 min) to visualize S phase proliferative cells, we examined the proliferation pattern in the palatal shelves. We could not observe a notable difference in number of EdU positive cells between WT and *Wiz^–/–^* embryos on histological sections at E13.5 (Figure 4A) and at E14.5 (Supplementary Figure 3A). There was no significant difference in ratio of proliferating to all cells in anterior and posterior regions in palatal shelves at E13.5 (Figure 4B) as well as at E14.5 (Supplementary Figure 3B). These results suggest that the cell proliferation levels are not affected at these developmental stages.

To examine the possible disruption of signaling pathways involved in palate development, we performed a RT-qPCR analysis (Supplementary Figure 3C) of WT and *Wiz^–/–^* whole palatal shelve tissue at E13.5. Our data show that signaling pathways such as Wnt (*Wnt5a*, *Axin1*, *Axin2*), Fgf (*Etv4*, *Etv5*, *Spry1*), Shh (*Gli*, *Shh*), Bmp (*Bmp2*, *Bmp4*), or TGF-β (*Tgf-*β) or Notch (*Hes1*, *Notch2*) are not significantly affected by Wiz ablation at this stage as well as expression of other cleft palate associated genes like *Msx2* or *Cdh1*.

The presence of G9a and GLP methylation complexes in developing palate provides a strong indication that Wiz might be responsible for regulation of the histone methylation code. We used antibodies against monomethylated and dimethylated Histone 3 Lysine 9 (H3-K9), which is the most common target of the G9a/GLP methylation complex, and compared the methylation pattern on histological sections from between E13.5 and E15.5.

**FIGURE 5:**
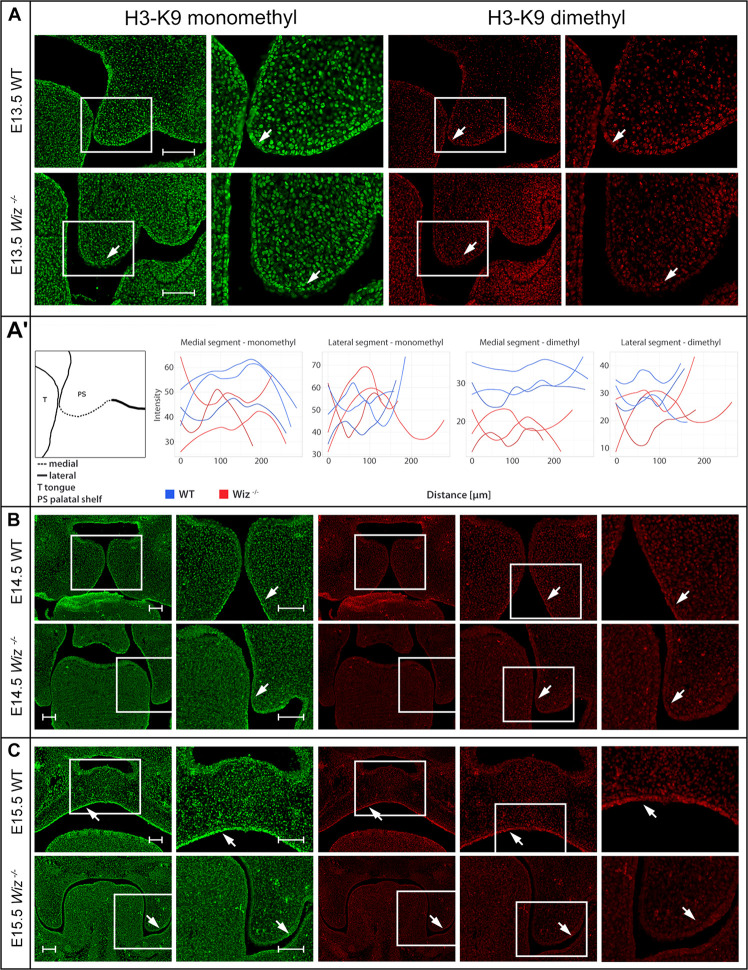
Wiz deficiency suppresses methylation pattern in palatal shelves. **(A)** Immunofluorescent double stained palatal sections of E13.5. **(A’)** Quantification of methylation marks in E13.5 palatal sections. On the left, schematic cartoon showing medial and lateral segments used for quantification. Rest of the panel consists of graphic visualization of spectral intensity quantification for medial and lateral segments of mono- and dimethylated H3-K9, respectively. **(B)** Immunofluorescent double stained palatal sections of E14.5 WT (upper panels) and *Wiz^–/–^* (lower panels) embryos for mono- and dimethylated H3-K9. Images show reduction of the signal in the *Wiz^–/–^* epithelium compared to positive areas in WT corresponding to the future oral surface of the shelves both before and after horizontalization. **(C)** Immunofluorescent double stained palatal sections of E15.5 WT (upper panels) and *Wiz^–/–^* (lower panels) embryos for mono- and dimethylated H3-K9. At this developmental stage, dramatic decrease of H3-K9 methylation levels was observed in palatal shelves of *Wiz^–/–^*, especially in the epithelial layer facing the tongue (white arrows). Second column for each staining displays detailed image from the first column labeled with white rectangle. In **(B,C)**, last column represents magnified images from the area labeled with white rectangle in previous column. Scale bar represents 100 μm and applies for all images.

Starting from the stage E13.5 we did not observe changes in monomethylated H3-K9 in any part of developing palatal shelves. However, we already observed loci specific diminution of the methylation signal from dimethylated H3-K9 in the *Wiz^–/–^* embryos (Figure 5A). The decrease of methylation marks was localized only in medial segment (part of developing palatal shelves) of future oral cavity epithelium in contrary to lateral segment (part of maxilla) (Figure 5A). The spectral intensity quantification supported these findings and also confirmed that the methylation mark immunostaining was comparable among the samples and lateral part of shelves.

Such phenomenon is observable also during later stages of palate development at E14.5 and E15.5 (Figures 5B,C). Despite the deregulation of methylation code at this stage cannot be considered as primary cause of palatal clefting, it is worth mentioning that such spatial-temporal diminution of methylation is observable over the most critical period of palatal development.

The medial segment corresponding to lateral part of palatal shelf epithelium seems to be the most affected and is corresponding to sites of the strongest expression of *Wiz* and *G9a/GLP* (Figure 3). Our data suggest the regulatory role of Wiz protein in temporo-spatial regulation of chromatin methylation correlates with delayed palatal shelves horizontalization. Nevertheless, the precise molecular mechanism of this action needs further examination, likely at single cell level and by unbiased transcriptomics.

## Discussion

It was previously shown (Ueda et al., 2006; Bian et al., 2015) that Wiz is a core subunit of the G9a/GLP methylation complex and important for the complex’s specificity. The G9a/GLP complex independently regulates both H3-K9 and DNA methylation, resulting in transcriptional silencing (Tachibana et al., 2008). Several studies have provided insight into the role of Wiz in mice, such as linking Wiz deficiency with behavioral phenotypes and anxiety (Isbel et al., 2016). It has been proposed that either WIZ or ZNF644 is sufficient for targeting the G9a/GLP complex to specific loci for H3-K9 methylation (Bian et al., 2015). On the other hand, multiple other G9a-associated molecules containing zinc finger motifs have been reported, such as PRDI-BF1 (Gyory et al., 2004) or ZNF217 (Banck et al., 2009). This may explain why the functional ablation of Wiz is not embryonically lethal as early as G9a or GLP themselves. Due to early embryonic lethality, Wiz*^*MommeD*30^* model (Daxinger et al., 2013) is not suitable for studying the function of Wiz gene in later embryonic development such as palate formation. Also, early lethality of some *Wiz^–/–^* embryos suggests presence of other than craniofacial defects creating opportunity for further developmental studies.

The data presented in this article support our hypothesis that Wiz deficiency causes disruption of methylation known to be driven by the G9a/GLP methylation complex, which we show is coexpressed with Wiz in the palatal shelf. In fact, our findings showed decreased H3-K9 methylation levels *in vivo*, which may result in developmental defects in mice fetuses limited to orofacial areas, especially epithelia of developing palatal shelves. These observations would be interesting to explore further with a precise genetic reporter system for analysis of changes in epigenetic regulations based on binding reporter to dimethylated H3-K9 or directly on Wiz activity on artificial promoter with reporter. It seems that specific population of epithelial cells on the medial edge of palatal process is affected. Highly specific molecular signature of these cells is likely crucial for the properly timed horizontalization and subsequent fusion of the palatal shelves. This hypothesis is supported by the fact that proliferation rate within the anterior and posterior region of the shelves is not changed despite emerging possible difference in shelf shape caused by the failure of the shelf horizontalization itself. Images used for presentation of this phenomenon are stage and section matched. *Wiz^–/–^* palatal shelves appear to have denser cells, which may not migrate properly and spread out to achieve the shape of WT palatal shelves. The inability to find alteration of expression between *Wiz^–/–^* and WT samples may be caused by the fact that whole palatal tissues were used for RT-qPCR, however, only small domain is affected by methylation defect, and thus single cell level investigation seems to be more appropriate. In addition, the relatively local effect of the mutation on the epithelium and subsequent presumable dysregulation of horizontalization timing explains why some of the *Wiz^–/–^* embryos display only incomplete cleft phenotype when the processes are able to reach each other but fail to fuse properly. Developmental delay of *Wiz^–/–^* palatal shelves horizontalization results in a loss of ability to fuse even if they manage to elevate. This observation is supported by Jin et al. (2008) reporting that loss of *Zfhx1a*, a transcription modulator of signaling pathways activated by members of TGF-β superfamily, leads to a cleft palate phenotype due to delayed palatal shelf elevation, while shelf size or cell proliferation rate is unchanged. By the E16.5, all of the *Zfhx1a* mutant palatal shelves were elevated above the tongue but remained separated. This 24–48 h developmental delay caused the palatal shelves to miss the window of competence to fuse (Jin et al., 2008). A similar situation was observed in retinoic acid (RA) teratogenic effect on palatal development. It has been shown that RA caused failure of palatal shelf elevation (Tang et al., 2016). Tang at al. showed that impaired palatal shelf elevation may be caused by delayed progress of cell condensation and the failure of the tongue withdrawal. No regional differences in cell proliferation ratio at E13.5 were reported in the RA induced cleft palate phenotype (Okano et al., 2007). On top of these findings, other observed phenotypes of E18.5 *Wiz ^–/–^* embryos such as cleft eyelids may be connected to the cleft palate phenotype as they share a similar developmental program, which consists of directional tissue growth followed by tissue fusion. Different types of eyelid defects were found among patients with cleft palate diagnosis (Anchlia et al., 2011). Similarly, shorter snout in mouse embryo may indicate more general alteration in growth dynamic of orofacial area, which can result in cleft phenotypes.

The connection between epigenetic chromatin modification and craniofacial defects is very complex and may be dependent or independent on DNA or histone modifications (Du et al., 2015). In recent years, altered DNA methylation was linked to non-syndromic cleft palate development (Alvizi et al., 2017; Gonseth et al., 2019), indicating that difference in methylation marks may contribute to a cleft palate phenotype. Interestingly also maternal exposure to RA inducing palate clefting is connected to dysregulated DNA methylation pattern in palatal shelves (Kuriyama et al., 2008). The need for proper epigenetic regulation was observed also on the effect of folic acid or choline, which are important co-factors for synthesis of the methyl donor group from S-adenosyl methionine. Both co-factors were linked to decreased risk of isolated non-syndromic cleft palate occurrence (Shaw et al., 2006; Wilcox et al., 2007) when overused during pregnancy. Together with genetic evidence that mutations in G9a are linked with Kleefstra syndrome, characterized by developmental delay and altered facial features (Willemsen et al., 2012), there is strong evidence that epigenetic regulation is involved in precise orchestration of timing in the critical developmental processes. Thus, palate development is one of the most vulnerable processes, because there is only a narrow window of competence when palatal shelves can fuse.

## Data Availability Statement

The datasets presented in this study can be found in online repositories. The names of the repository/repositories and accession number(s) can be found in the article/Supplementary Material.

## Ethics Statement

The animal study was reviewed and approved by the Animal Care and Use Committee of the Institute of Molecular Genetics.

## Author Contributions

IB performed the experiments, analyzed the data, and wrote the manuscript. KS produced the mice and initiated the project. MP performed the experiments and analyzed the data. IMB generated the mouse model. JP designed the experiments, analyzed the data, wrote the manuscript, and supervised the research. RS wrote the manuscript and supervised the research. All authors contributed to the article and approved the submitted version.

## Conflict of Interest

The authors declare that the research was conducted in the absence of any commercial or financial relationships that could be construed as a potential conflict of interest.

## Supplementary Material

Supplementary Figure 1 Probe design for *in situ* hybridization. **(A)** Plasmid maps of G9a- pGEM^®^-T plasmids used for probe preparation and sequence of interest. **(B)** Plasmid maps of GLP- pGEM^®^-T plasmids used for probe preparation and sequence of interest. **(C)** Plasmid maps of Wiz- pGEM^®^-T plasmids used for probe preparation and sequence of interest. Colors in text are corresponding to colors in the plasmid map. Restriction sites are written in red, primers sequences written in bold and underlined. Created with BioRender.com. **(D)** G9a, GLP, and Wiz sense probes generated as a negative control for *in situ* images.

Supplementary Figure 2 Generation of Wiz deficient mouse model. **(A)** Schematic cartoon visualizing targeting exon 4 of Wiz gene by CRISPR/Cas9 resulting in 32 nucleotide deletion, frame-shift mutation and premature stop codon. Created with BioRender.com. **(B)** Western blot analysis from *Wiz^–/–^*, *Wiz*^±^, and WT embryonic lysate showing protein Wiz sized 130 kDa in WT sample, lower amount in heterozygous sample and none present in KO sample. **(C)** Localization of SDS-Page gel cutout for Mass Spectrometry. **(D)** Mass spectrometry analysis from gel cutouts and graphic visualization of logarithmic number of particles for protein Wiz present in WT sample. Contrary to that, no Wiz protein detected in *Wiz^–/–^* sample.

Supplementary Figure 3 Wiz deficiency does not affect number of proliferative cells. **(A)** EdU labeling shows no difference in proliferation levels in palatal shelves between WT (upper panels) and KO (lower panels) palatal sections at E14.5. Scale bar represents 100 μm and applies for all images. White rectangles on the right side panels show the area that is presented magnified on the left side panels respectively. **(B)** Quantification of EdU labeled proliferating cells shows no difference in ratio of proliferating cells between genotypes at E14.5 in anterior (*p* = 0.305) and posterior regions (*p* = 0.468) of palatal shelves. *n* = 3 *Wiz^–/–^* vs. 3 WT embryos, with five sections from anterior and five sections from posterior part of shelves for each embryo. **(C)** Expression levels of representative genes from signaling pathways involved in palatogenesis do not change between genotypes. *n* = 4 *Wiz^–/–^* and WT samples. Expression levels of the genes of interest were normalized to levels of Rpl19 and are presented as levels relative to wild type (set as 1).

Supplementary Methods Supplementary methods for SDS-PAGE and immunoblotting, in gel digestion, nLC-MS 2 Analysis, Data analysis, and Quantitative real time reverse transcription polymerase chain reaction (qRT-PCR).
