# Supplementary figures and images for: Loss of Wiz Function Affects Methylation Pattern in Palate Development and Leads to Cleft Palate
Source: Front Cell Dev Biol. 2021 Jun 2;9:620692. doi: 10.3389/fcell.2021.620692 (PMC8206640; doi:10.3389/fcell.2021.620692)

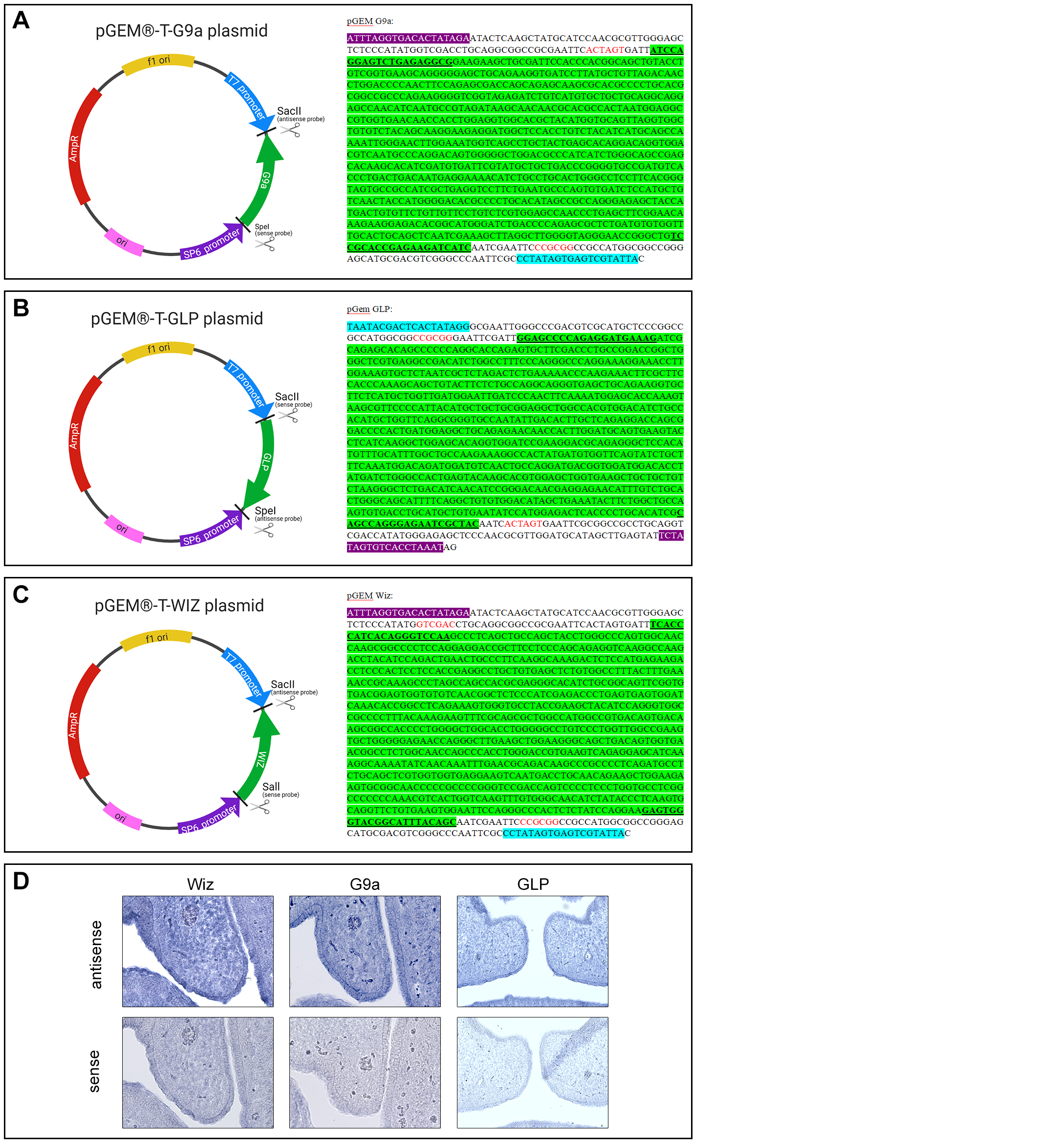

Supplement: Supplementary Figure 1 — Probe design for in situ hybridization. (A) Plasmid maps of G9a- pGEM®-T plasmids used for probe preparation and sequence of interest. (B) Plasmid maps of GLP- pGEM®-T plasmids used for probe preparation and sequence of interest. (C) Plasmid maps of Wiz- pGEM®-T plasmids used for probe preparation and sequence of interest. Colors in text are corresponding to colors in the plasmid map. Restriction sites are written in red, primers sequences written in bold and underlined. Created with BioRender.com. (D) G9a, GLP, and Wiz sense probes generated as a negative control for in situ images. [file Image_1.TIF]

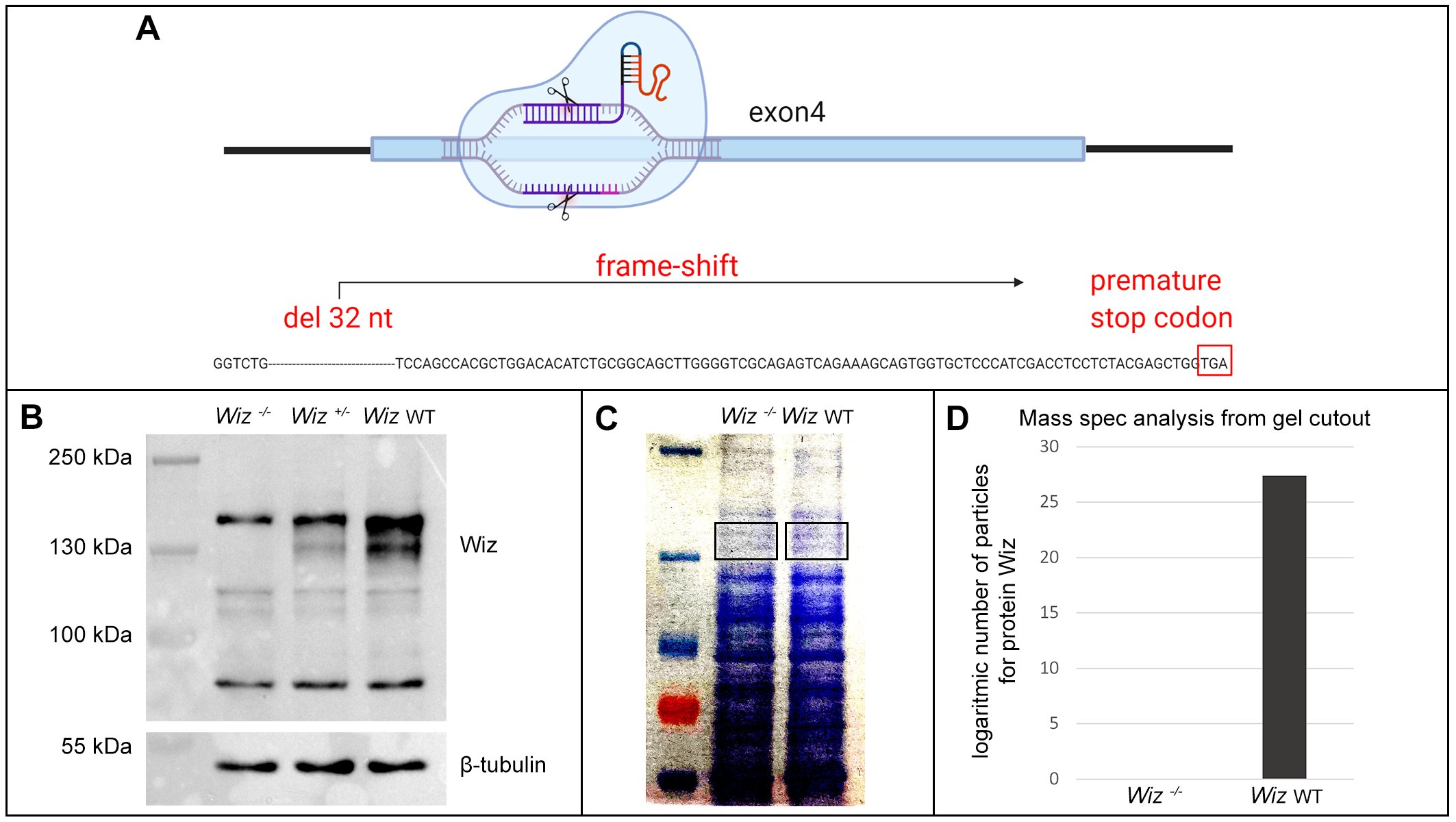

Supplement: Supplementary Figure 2 — Generation of Wiz deficient mouse model. (A) Schematic cartoon visualizing targeting exon 4 of Wiz gene by CRISPR/Cas9 resulting in 32 nucleotide deletion, frame-shift mutation and premature stop codon. Created with BioRender.com. (B) Western blot analysis from Wiz–/–, Wiz±, and WT embryonic lysate showing protein Wiz sized 130 kDa in WT sample, lower amount in heterozygous sample and none present in KO sample. (C) Localization of SDS-Page gel cutout for Mass Spectrometry. (D) Mass spectrometry analysis from gel cutouts and graphic visualization of logarithmic number of particles for protein Wiz present in WT sample. Contrary to that, no Wiz protein detected in Wiz–/– sample. [file Image_2.TIF]

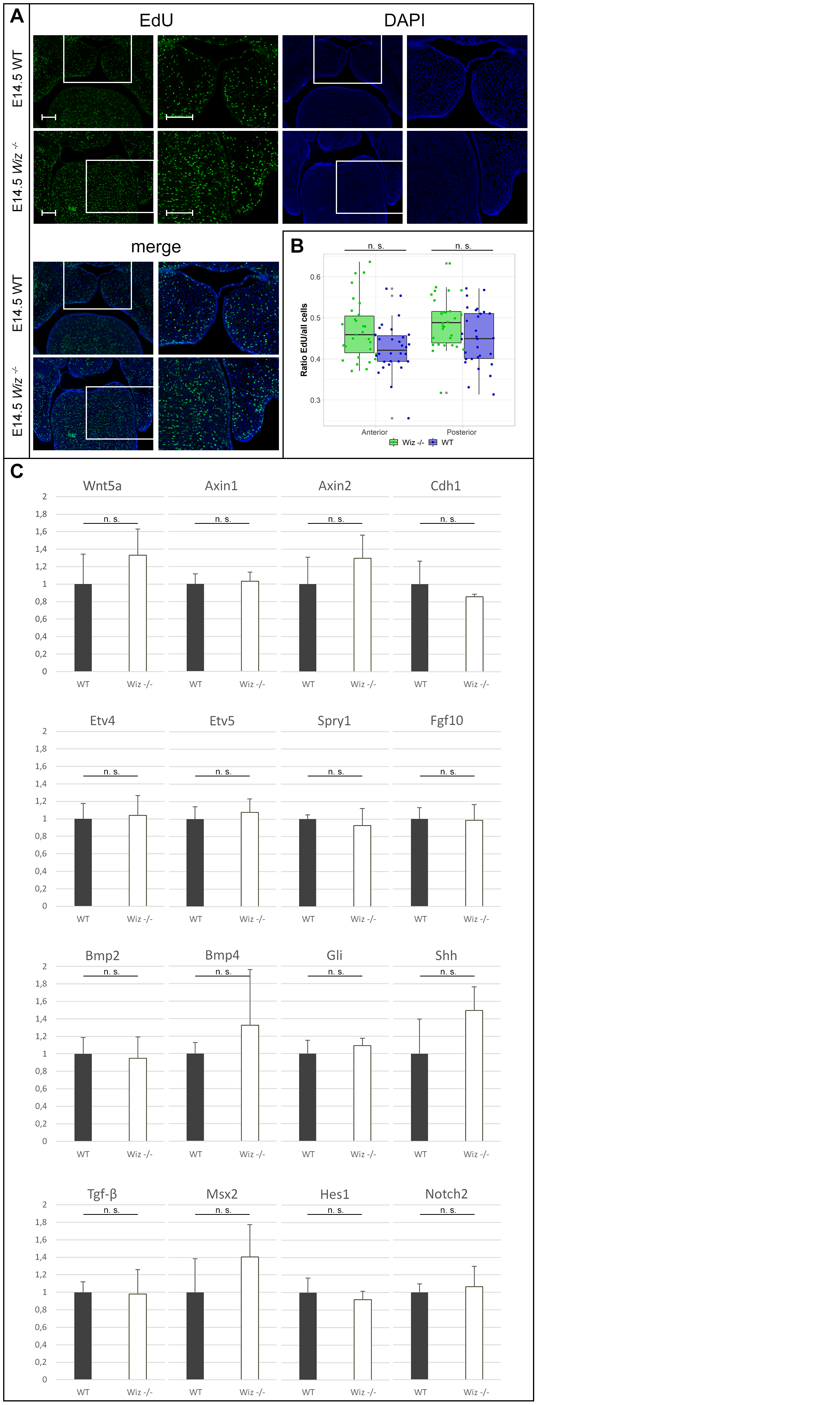

Supplement: Supplementary Figure 3 — Wiz deficiency does not affect number of proliferative cells. (A) EdU labeling shows no difference in proliferation levels in palatal shelves between WT (upper panels) and KO (lower panels) palatal sections at E14.5. Scale bar represents 100 μm and applies for all images. White rectangles on the right side panels show the area that is presented magnified on the left side panels respectively. (B) Quantification of EdU labeled proliferating cells shows no difference in ratio of proliferating cells between genotypes at E14.5 in anterior (p = 0.305) and posterior regions (p = 0.468) of palatal shelves. n = 3 Wiz–/– vs. 3 WT embryos, with five sections from anterior and five sections from posterior part of shelves for each embryo. (C) Expression levels of representative genes from signaling pathways involved in palatogenesis do not change between genotypes. n = 4 Wiz–/– and WT samples. Expression levels of the genes of interest were normalized to levels of Rpl19 and are presented as levels relative to wild type (set as 1). [file Image_3.TIF]
