# Supplementary material for: Loss of Wiz Function Affects Methylation Pattern in Palate Development and Leads to Cleft Palate
Source: Front Cell Dev Biol. 2021 Jun 2;9:620692. doi: 10.3389/fcell.2021.620692 (PMC8206640; doi:10.3389/fcell.2021.620692)
Supplement: Supplementary Methods — for SDS-PAGE and immunoblotting, in gel digestion, nLC-MS 2 Analysis, Data analysis, and Quantitative real time reverse transcription polymerase chain reaction (qRT-PCR). [file Data_Sheet_1.pdf]

SUPPLEMENTARY METHODS

SDS-PAGE and immunoblotting

Embryonic tissue was harvested into RIPA buffer (150mM NaCl, 1% Nonidet P-40, 0.5% Sodium deoxycholate, 0.1% SDS, 1mM EDTA, 50mM Tris/HCl pH8) with phosphatase and protease inhibitors PhosSTOP (Roche, Germany) and cOmplete Tablets, mini (Roche, Germany) and dissociated in Tissue Lyser II (Qiagene). Concentration of proteins was estimated by the BCA method (Pierce Biotechnology Inc., Rockford, USA). Before separation by 7% SDS-PAGE, 100 mM DTT and 0.01% bromophenol blue were added to the cell lysates. The same protein amount (20 μg) was loaded into each well. After electrophoresis, proteins were electrotransferred from the gel onto a nitrocellulose membrane using the wet transfer method and detected by specific antibodies WIZ Antibody (Novus Biologicals, NBP1-80586) at 1:1000 dilution and β-tubulin (Santa Cruz, cs-2146s. Detection was performed by SuperSignal West Pico PLUS Chemiluminiscent Substrate (Thermo Scientific, #34580).

In gel digestion

Individual bands containing proteins of interest were excised from Coomasie stained SDS PAGE gel using a razor blade and cut into small pieces (aprox. 1 mm x 1 mm). Bands were destained by sonication for 30 min in 50% acetonitrile (ACN) and 50mM ammonium bicarbonate (ABC). After destaining, solution was removed and gels were dried in ACN. Disulfide bonds were reduced using 10mm DTT in 100mM ABC at 60°C for 30 min. After that, samples were again dried with ACN and free cystein residues were blocked using 55mM iodoacetamide in 100mM ABC for 10 min at room temparature in dark. Samples were dried thoroughly and digestion buffer (10% ACN, 40mM ABC and 13 ng/μl trypsin) was added to cover gel pieces. Proteins were digested at 37 °C overnight. After digestion, 150 μl of 50% ACN with 0,5% formic acid was added and sonicated for 30 min. Supernatant containing peptides was replaced to a new micro centrifuge tube and another 150 μl of elution solution was added and sonicated for 30 min. This solution was removed, combined with previous solution and dried using Speedvac. Dried peptides were reconstituted in 2% ACN with 0,1% TFA and injected into Ultimate 3000 Nano LC coupled to Orbitrap Fusion.

nLC-MS 2 Analysis

Nano Reversed phase columns (EASY-Spray column, 50 cm x 75 µm ID, PepMap C18, 2 µm particles, 100 Å pore size) were used for LC/MS analysis. Mobile phase buffer A was composed of water and 0.1% formic acid. Mobile phase B was composed of acetonitrile and 0.1% formic acid. Samples were loaded onto the trap column (C18 PepMap100, 5 µm particle size, 300 µm x 5 mm, Thermo Scientific) for 4 min at 18 µl/min loading buffer was composed of water, 2% acetonitrile and 0.1% trifluoroacetic acid. Peptides were eluted with Mobile phase B gradient from 4% to 35% B in 120 min. Eluting peptide cations were converted to gas-phase ions by electrospray ionization and analyzed on a Thermo Orbitrap Fusion (Q-OT- qIT, Thermo Scientific). Survey scans of peptide precursors from 350 to 1400 m/z were performed in orbitrap at 120K resolution (at 200 m/z) with a 5 × 105 ion count target. Tandem MS was performed by isolation at 1,5 Th with the quadrupole, HCD fragmentation with normalized collision energy of 30, and rapid scan MS analysis in the ion trap. The MS2 ion count target was set to 104 and the max injection time was 35 ms. Only those precursors with charge state 2–6 were sampled for MS2. The dynamic exclusion duration was set to 45 s with a 10 ppm tolerance around the selected precursor and its isotopes. Monoisotopic precursor selection was turned on. The instrument was run in top speed mode with 2 s cycles (Hebert, Richards et al., 2014).

Data analysis

All data were analyzed and quantified with the MaxQuant software (version 1.6.3.4) (Cox & Mann, 2008). The false discovery rate (FDR) was set to 1% for both proteins and peptides and we specified a minimum peptide length of seven amino acids. The Andromeda search engine was used for the MS/MS spectra search against the *Mus musculus* database (downloaded from Uniprot on January 2021, containing 17 059 entries). Enzyme specificity was set as C-terminal to Arg and Lys, also allowing cleavage at proline bonds and a maximum of two missed cleavages. Dithiomethylation of cysteine was selected as fixed modification and N- terminal protein acetylation and methionine oxidation as variable modifications. The "match between runs" feature of MaxQuant was used to transfer identifications to other LC-MS/MS runs based on their masses and retention time (maximum deviation 0.7 min) and this was also used in quantification experiments. Quantifications were performed with the label-free algorithm in MaxQuant (Cox et al., 2014). Data analysis was performed using Perseus 1.6.1.3 software (Tyanova et al., 2016).

Quantitative real time reverse transcription polymerase chain reaction (qRT-PCR)

Total RNA from E13.5 palatal shelves was isolated using RNeasy Mini Kit (Qiagen, USA) according to the manufacturer's protocol. 4 embryos from Wiz$^{-/-}$ and 4 control littermates were used for the analysis. All samples were run in technical triplicates. cDNA was synthesized from 1000 ng of total RNA with Random Primers and Oligo (dT)15 primer (Promega, USA) using M-MLV Reverse Transcriptase and buffer (Promega, USA), RNasin® Ribonuclease Inhibitor (Promega, USA) and dNTP, Deoxynucleotide Mix 10 mM (Sigma, Germany). qRT-PCR was performed in The LightCycler® 480 (Roche, Germany) using LightCycler® 480 SYBR Green I Master (Roche, Germany).

The following sets of primers were used:

Wnt5a: FW: 5'-CAAATAGGCAGCCGAGAGAC-3',RV: 5'-CTCTAGCGTCCACGAACTCC-3'
Axin1: FW: 5'-GCTGACACGCTATCCCTTAC-3', RV: 5'-TACTCTCCTGCATCTCCCTT-3'
Axin2: FW: 5'-GTCCGCCTCCCCAAAG-3', RV: 5'-TCCAGTTCCTCTCAGCAATC-3'
Cdh1: FW: 5'-GTCCTGCCAATCCTGATGAA-3', RV: 5'-GAACACCAACAGAGAGTCGT-3'
Etv4: FW: 5'-AGCAGGAAGCCACCACT-3', RV:ATGGCGATTTGTCTGGGG-3'
Etv5: FW: 5'-CCCGAGATTACTGTGCTGAC-3', RV: 5'-ATGGCTGCTGGAGAAATACC-3'
Gli1: FW: 5'-GAGGTTGGGATGAAGAAGCA-3', RV: 5'-CATTGGATTGAACATGGCGT-3'
HES1: FW: 5'-CCAAGCTAGAGAAGGCAGAC-3', RV: 5'-GGTATTTCCCCAACACGCTC-3'
Bmp2: FW: 5'-AGCAGCAACACTAGAAGACAG-3',RV: 5'-TGTGGAGACTCTCTCAATGGA-3'
Bmp4: FW: 5'-GAACAAACTTGCTGGAAAGGC-3', RV: 5'-TCATTGCAGCTTTCTAGAGGTC-3'
Spry1: FW: 5'-GCCCAGTATCTGTGAAGGAC-3', RV: 5'-ATACCAGGGGCAAATCAGAC-3'
Msx2: FW: 5'-CTCGGTCAAGTCGGAAAA-3', RV: 5'-GTGCAGGTGGTGGGG-3'
Shh: FW: 5'-GAATCCAAAGCTCACATCCAC-3', RV: 5'-CGTAAGTCCTTCACCAGCTTG-3'

TGF-β: FW: 5’-ACTGATACGCCTGAGTGGCT-3’, RV: 5’-TTGCTGTCACAAGAGCAGTGA-3’

Notch2: FW: 5’-GCCACCTGCAATGACTTCA-3’, RV: 5’-CACTCGTCCACTTCATACTCAC-3’

Fgf10: FW: 5’- CAACTCCGATTTCCACTGATGT-3’, RV: 5’-GCTGTTCTCCTTCACCAAGT-3’

Rpl19 FW: 5’-ATGAGACCAATGAAATCGCC-3’, RV: 5’-CACAGGCTTGCGGATGA-3’

The relative quantity of cDNA was estimated by the ΔΔCt method (Livak & Schmittgen, 2001). Expression levels of the genes of interest were normalized to levels of Rpl19 and are presented as levels relative to wild type (set as 1).
